# Atypical course of a caesarean scar pregnancy

**DOI:** 10.1007/s00404-022-06697-9

**Published:** 2022-07-15

**Authors:** Katharina Schlammerl, Stefan Kommoss, Bernhard Krämer, Markus Hoopmann, Cornelia Bachmann

**Affiliations:** Department of Women’s Health Tübingen, Eberhard Karls University Tübingen, 72076 Tübingen, Germany

**Fig. 1:**
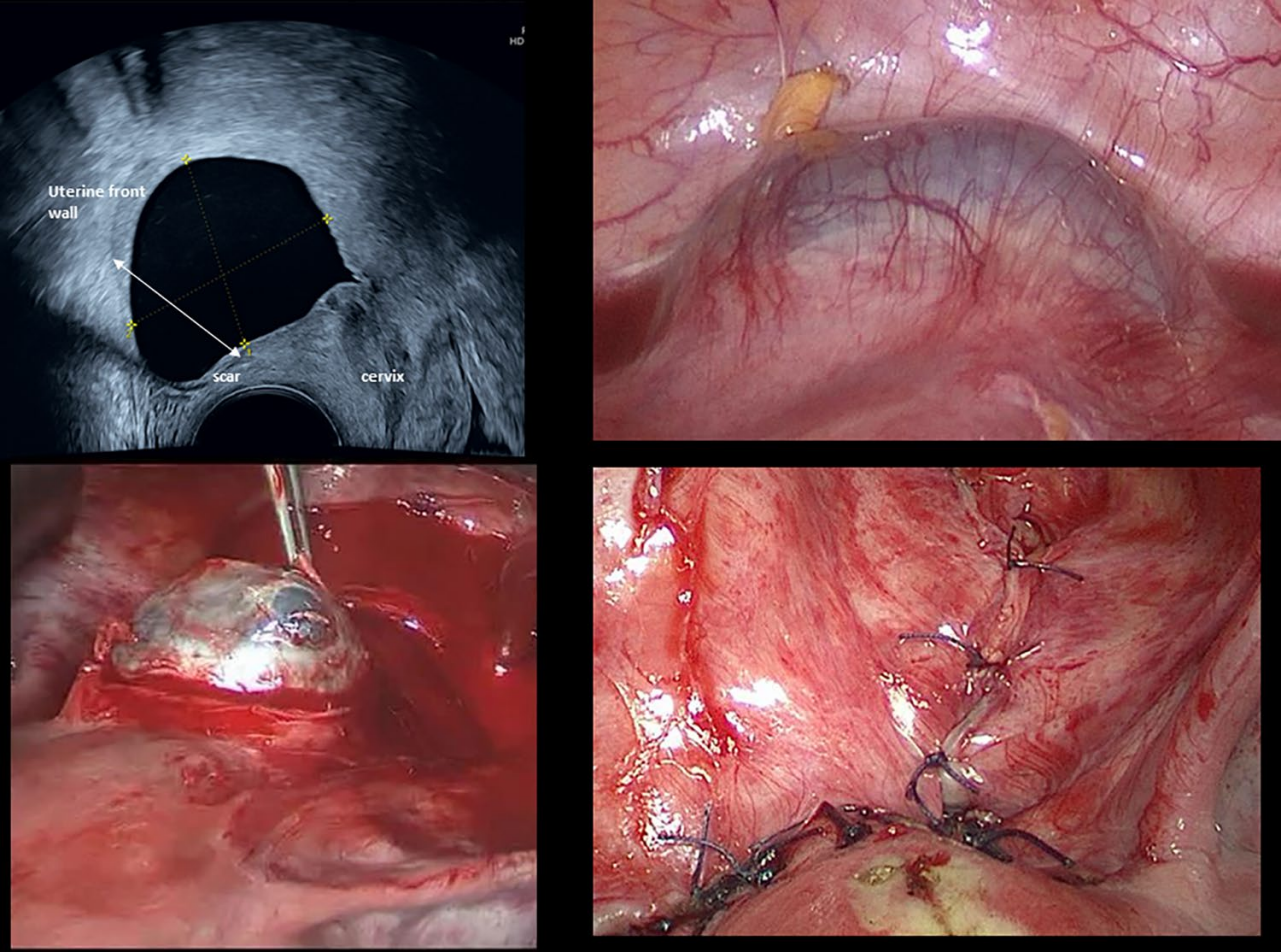
**a** Transvaginal ultrasound image of caesarean scar pregnancy with a cystic structure of 4 × 3 × 3 cm of a non-vital pregnancy at 15th week of gestation. Anatomic structures are labelled. **b** By laparoscopy: detection of the cystic structure sized 4 × 3 × 3 cm at the isthmo-cervical junction. **c** Situs of open surgery with demonstration of the above mentioned cystic structure at the isthmo-cervical junction. **d** Uterine reconstruction after removing the lesion

The 36-year old patient presented first with a caesarean scar pregnancy (vital) at 7 weeks of gestation. In her medical history, she had 2 miscarriages (10/21 and 12/21) without curettage and a caesarean section (Misgav-Ladach) in 2019. Ultrasound revealed a vital pregnancy in the caesarean scar while large parts of the trophoblast have grown into it reaching up to the serosa (β-HCGlevel: 26873U/l). Due to the risk of bleeding, a therapy with methotrexate was initiated and was administered intravenously, mifepristone was applied orally once [1]. The sonographic check-up (9th gestational week) detected a non-vital pregnancy with a chorionic cavity of 2.8 cm. Three weeks later and after application of methotrexate (2 courses) the sonographic check-up revealed the embryonic structures in regression and a decreasing perfusion by Doppler sonography. At the same time, a discrete progression of the chorionic cavity was noted (size: 3.4 × 3.2 × 2.3 cm), while the β-HCGlevel dropped to 2348U/l. Another 2 weeks later, the ultrasound check-up detected no more embryonic structures, a significantly reduced blood flow and a thinned trophoblast ring. β-HCGlevel dropped down (429U/l). Despite the serologically and sonographically visible reduction in vital tissue, the chorionic cavity increases in volume at the isthmo-cervical junction with a size of 4 × 3 × 3 cm. At 16th gestational week and after a total of three courses of methotrexate (i.v.), no further progression of the cystic structure was detected in the ultrasound check-up (β-HCG level: 116U/l) (Fig. 1a) [2]. Due to the progression of the cystic structure despite methotrexate administration, a curettage with simultaneous laparoscopic control was scheduled [3, 4]. The isthmo-cervical lesion was confirmed laparoscopically (Fig. 1b) [5]. Due to the increased risk of bleeding, the lesion was removed by laparotomy (Fig. 1c and d) followed by uterine reconstruction (blood loss: 400 ml) [6]. The histology report revealed diagnosis. The patient was discharged home a few days later with no complaints (Fig. 1).
